# The Impact of Pulmonary Arterial Pressure on Exercise Capacity in Mild-to-Moderate Cystic Fibrosis: A Case Control Study

**DOI:** 10.1155/2012/252345

**Published:** 2012-07-29

**Authors:** Katerina Manika, Georgia G. Pitsiou, Afroditi K. Boutou, Vassilis Tsaoussis, Nikolaos Chavouzis, Marina Antoniou, Maria Fotoulaki, Ioannis Stanopoulos, Ioannis Kioumis

**Affiliations:** ^1^Pulmonary Department, Aristotle University of Thessaloniki, “G. Papanikolaou” General Hospital, Exohi, 57010 Thessaloniki, Greece; ^2^Respiratory Failure Unit, Aristotle University of Thessaloniki, “G. Papanikolaou” General Hospital, Exohi, 57010 Thessaloniki, Greece; ^3^4th Department of Pediatrics, Aristotle University of Thessaloniki, “Papageorgiou” General Hospital, 56429 Thessaloniki, Greece

## Abstract

*Background*. Pulmonary hypertension (PH) is an often complication of severe cystic fibrosis (CF); however, data on the presence and impact of pulmonary vasculopathy in adult CF patients with milder disease, is very limited. *Aim*. To investigate, for the first time, the impact of systolic pulmonary arterial pressure (PASP) on maximal exercise capacity in adults with mild-to-moderate cystic fibrosis, without PH at rest. *Methods*. This is a Case Control study. Seventeen adults with mild-to-moderate CF, without PH at rest (cases) and 10 healthy, nonsmoking, age, and height matched controls were studied. All subjects underwent maximal cardiopulmonary exercise testing and echocardiography before and within 1 minute after stopping exercise. *Results*. Exercise ventilation parameters were similar in the two groups; however, cases, compared to controls, had higher postexercise PASP and decreased exercise capacity, established with lower peak work rate, peak O_2_ uptake, anaerobic threshold, and peak O_2_ pulse. Furthermore, the change in PASP values before and after exercise was strongly correlated to the parameters of exercise capacity among cases but not among controls. *Conclusions*. CF adults with mild-to-moderate disease should be screened for the presence of pulmonary vasculopathy, since the elevation of PASP during exercise might contribute to impaired exercise capacity.

## 1. Introduction

Exercise impairment in cystic fibrosis (CF) is well established and a variety of determinants, such as pulmonary and nutritional factors, muscle dysfunction and deconditioning, have been studied in this direction [1–4]. It seems that the factors which are limiting exercise tend to vary across disease stages; ventilatory impairment is probably the major factor limiting exercise in severe disease, while nonpulmonary factors seem to be related to reduced exercise capacity in mild and moderate disease [4].

Pulmonary hypertension (PH), which is a common determinant of exercise capacity in patients with respiratory disorders [5], is an often complication of CF. PH is observed in 20–65% of adult CF patients with severe disease [6–10], and it has been associated with increased mortality [6, 11]. However, data on its frequency and impact among patients with milder disease are limited. Although adult CF patients with mild-to-moderate disease achieve maximum exercise without generally reaching ventilatory limitation [4], the potential effect of pulmonary vasculopathy on exercise capacity, in this patient population, has not yet been clarified.

In this study we hypothesized that pulmonary vascular disease contributes to the exercise intolerance of adult CF patients with mild-to-moderate disease, without PH at rest. Under this scope we conducted a case-control study, utilizing maximal cardiopulmonary exercise testing (CPET), in order to investigate, for the first time in literature, the impact of post exercise PASP on this patient population.

## 2. Methods

### 2.1. Subjects

The study followed a case-control design. Seventeen adult CF patients with mild-to-moderate disease constituted the group of cases and 10 healthy nonsmoking volunteers matched for age and height, constituted the group of controls. All CF patients were regularly attending the outpatient CF clinic of “G. Papanikolaou” Hospital, were in stable clinical condition and were life-long nonsmokers. Mild pulmonary disease was defined by the presence of FEV_1_ >65% of predicted, and moderate disease by the presence of ≤40% FEV_1_ ≤65% of predicted [6]. Patients who presented with an exacerbation of the disease, that is increased sputum production, purulence, or dyspnea with or without systemic symptoms, combined with a 10% or more reduction in patient's forced expiratory volume in 1 second (FEV_1_) usual value, were excluded from the study. Patients who were hospitalized or required per os antibiotics during the last 2 months prior to the study, received domiciliary oxygen therapy, or presented with any other condition affecting exercise capacity, were also excluded. Ethical approval for the study protocol was received from the “G. Papanikolaou” Hospital Scientific Committee and informed consent was provided by all participants.

### 2.2. Study Protocol

During the baseline visit, all CF patients were clinically assessed by the same physician, who documented their Schwachmann score (SS). An arterial blood sample was obtained for both cases and controls, while all participants underwent pulmonary function testing. Forced vital capacity (FVC) and FEV_1_ were measured utilizing an electronic spirometer (Wright ventilometer, Clement Clarke International Ltd., England), according to the American Thoracic Society recommendations [12].

All participants visited again the CF clinic within one week and underwent a complete transthoracic echocardiographic study, including 2D, pulsed and continuous-wave Doppler and colour flow imaging using a HD7 cardiac ultrasound system (Philips medical system, Andover, MA, USA). Standard two-dimensional (2D) and colour flow Doppler images were obtained using the parasternal long and short axis and apical views. PASP was estimated by calculating the maximal velocity of the tricuspid regurgitant jet and by further using the Bernoulli equation and then adding to this value an estimated right atrial pressure based on both the size of the inferior vena cava and the change in diameter of this vessel during respiration. PH was defined as a PASP >35 mmHg at rest [13]. Left ventricular dimensions, ejection fraction, and cardiac index were obtained by previously recommended techniques [14].

A maximal exercise capacity test was then performed on a cycle ergometer (medical graphics), under continuous monitoring of heart rate (HR), oxygen saturation (SpO_2_), and a 12-lead electrocardiogram, while blood pressure (BP) measurements were obtained every two minutes using a standard-cuff mercury sphygmomanometer. A ramp protocol was used with an incremental rate of 20 Watts·min^−1^ for controls and of 10–20 Watts·min^−1^ for cases according to disease severity and estimated fitness, aiming for the test to last approximately 10–12 minutes [15]. Each test was preceded by 3 min of resting to enable subjects to achieve steady state conditions for HR, SpO_2_, BP, and gas exchange variables, and of 2 min of unloaded cycling. Patients and controls were asked to score their sense of dyspnea and muscle fatigue using Borg scale, every 2 minutes during the test.

Gas exchange values and exercise parameters were collected breath-by-breath and computer-averaged over 10-second intervals. Anaerobic threshold was calculated by the V slope method, as previously described [15]. The following exercise parameters were recorded: maximal work rate (WR peak), peak oxygen uptake (VO_2_ peak), oxygen uptake at anaerobic threshold (AT), peak oxygen pulse (VO_2_ peak/HR), ventilatory equivalent for carbon dioxide at AT (VE/VCO_2_), maximal ventilation VEmax, peak heart rate (HR), maximum minute ventilation to maximum voluntary ventilation ratio (VEmax/MVV), and breathing reserve (BR). The MVV was calculated as 40 × FEV_1_ and the breathing reserve as MVV-VEmax. Ventilatory limitation was defined as (VEmax/MVV) × 100 > 85% or BR <11 lit [15, 16].

Within 1 minute after the completion of the exercise testing, a second echocardiogram was performed by the same investigator, following the same protocol and focusing on postexercise tricuspid regurgitation velocity.

### 2.3. Data Analysis

Data analysis was conducted using the Statistical Package for Social Sciences (SPSS) for Windows 2000XP, release 17.0. Normal predicted values for CPET parameters were calculated using standard equations [15, 16]. The Shapiro-Wilk test of normality was used to assess the normal or not distribution of data. Student's *t*-test for independent samples was used to compare CPET parameters between cases and controls, while the Mann-Whitney the *U*-test was applied to compare CPET parameters between CF patients with low (≤35 mmHg) and high (>35 mmHg) postexercise PASP. ΔSPAP was calculated as follows: PASP after exercise − PASP at rest. Pearson correlation coefficient (*r*) was used to assess potential correlations between ΔPASP and CPET parameters in cases and controls. A *P* value <0.05 was considered significant.

## 3. Results

Summary characteristics of cases and controls are shown in Tables 1 and 2. During rest, none of the CF patients suffered from PH and no difference was noted in PASP or any other echocardiographic measurement between the two groups. However, PASP immediately after exercise was significantly higher in the group of cases compared to controls (31.5 versus 25.8, *P* = 0.041).

Both cases and controls stopped exercise because of fatigue. At the end of exercise mean Borg scale for fatigue was approximately 8 and mean Borg scale for dyspnea was 6 for both groups. Respiratory exchange ratio (RER) at peak exercise was >1.1 for all subjects. During maximal CPET, CF patients presented limited exercise capacity, compared to controls, as established by lower WR, VO_2_ peak, AT, and oxygen pulse (Table 2). However, no participant presented with respiratory limitation. Although the absolute value of MVV-VEmax was higher among cases compared to controls, VEmax/MVV% predicted did not differ between the two groups and even though BR was significantly lower among cases (Table 2), it was higher than 11 liters in all participants. Oxygen saturation at peak exercise was also similar between the two groups (Table 2).

After exercise, 5 cases presented with PASP >35 mmHg and the rest 12 cases with PASP ≤35 mmHg, while all controls had PASP ≤35 mmHg. No difference was noted between the two CF patient groups with and without exercise-induced PH, regarding FEV_1_% predicted and FVC% predicted values (data not shown). Those CF patients with post exercise PASP >35 mmHg exhibited lower WR peak% predicted, VO_2_ peak% predicted, VO_2_/HR% predicted, and SpO_2_ peak, and a trend for higher VE/VCO_2_@AT, compared to the rest of CF patients. However, none of the parameters indicative of ventilatory limitation during exercise, that is VEmax, VEmax/MVV and BR differed between CF patients with and without postexercise PH (Table 3).

In cases, but not in controls, ΔSPAP established an inverse, strong correlation to several parameters of exercise capacity, that is, WR peak (watts and % predicted), VO_2_ peak (mL/kg∗min and % predicted), oxygen pulse (mL/kg∗beats and % predicted), and SpO_2_ peak (Table 4). ΔSAP was also strongly correlated to Schwachman score (Spearman rho = −0.698, *P* = 0.002) in the group of cases. On the contrary, neither pulmonary function testing parameters, nor any variable indicative of ventilatory limitation during exercise was correlated to ΔSPAP, in any of the groups (data not shown).

## 4. Discussion

The main findings of our study are (a) patients with mild-to-moderate CF without PH at rest, exhibit higher postexercise PASP and lower exercise capacity compared to controls, without reaching ventilatory limitation, (b) exercise impairment and dyspnea are probably more pronounced among CF patients with higher (≥35 mmHg) postexercise PASP, and (c) ΔPASP is inversely correlated with maximal work rate and oxygen uptake in cases, but not in controls.

The association of PH and exercise tolerance in mild-to-moderate CF is far from clear. Montgomery et al. have reported the case of a CF adult patient with severe lung disease and pulmonary hypertension that increased significantly after exercise and improved with sildenafil treatment [17]. Although recently published data demonstrate that CF patients suffer from endothelial dysfunction and defective dilatation of pulmonary vessels during exercise [18], in another study the estimated rest PASP was not correlated to submaximal exercise capacity among CF patients with both severe and moderate disease [19]. However, there is no data regarding the exercise-induced increase of pulmonary artery pressure and its possible impact on maximum exercise tolerance, in patients with less severe disease and no evident pulmonary vasculopathy at rest.

In this study, a group of mild-to-moderate CF patients without PH at rest exhibited a higher postexercise PASP, a lower exercise capacity and a higher VE/VCO_2_ ratio at anaerobic threshold compared to controls, although both groups terminated exercise due to fatigue, without presenting respiratory limitation. VE/VCO_2_ is considered to be a noninvasive marker of pulmonary vascular resistance [20] and previous studies have reported a significant increase in the VE/VCO_2_ slope, during exercise, both in CF patients [1] and in patients with severe PH [21]. During maximal CPET, both dyspnea and exercise limitation were even more pronounced among patients with higher (>35 mmHg) postexercise PASP values, compared to the rest of the patients. Furthermore, ΔPASP values correlated to peak work rate, peak O_2_ uptake, O_2_ pulse and SpO_2_ at peak exercise only in the group of cases, while no correlation was noted to any measurement of ventilation during exercise. These data indicate that in CF patients with less severe disease, pulmonary circulation could be defective, resulting to impaired exercise capacity, regardless of the patients' respiratory reserve.

As in several chronic respiratory diseases, exercise capacity in adult CF patients could also be influenced by suboptimal nutritional status and muscle dysfunction [22, 23]. Malnutrition results, through a loss of muscle mass, to a reduction in every day activities and to peripheral muscle deconditioning [23]. The current study was not designed to control for these confounders, so their specific impact on exercise limitation could not be assessed. However, patients and controls weighted the same, since they were height-matched and had the same BMI, which indicates that their nutritional status was similar. Moreover, there was no difference in peak Borg fatigue score neither between patients and controls, nor between patients with and without exercise induced PH, indicating a similar peripheral muscle effort. These results come to an agreement with a previous study where differences in Borg scores of muscle effort and lactic acid were noted only in the group of CF patients with severe respiratory limitation and not among those with mild and moderate disease [4]. Future studies are needed to assess the exact impact of pulmonary vasculopathy on exercise capacity in these patient group, independently of nutritional status and muscle dysfunction.

There are certain limitations in this study. The number of participants who were included was quite small. However, our findings regarding exercise performance are very similar to the ones from larger cohorts [2]. Moreover, although the “gold standard” for measurement of pulmonary artery pressure remains right heart catheterization, Doppler echocardiography has proved to be an easily accessible, noninvasive alternative in previous studies [24]. Another limitation is that PASP is very much affected by cardiac output, so a higher postexercise PASP might reflect not a pulmonary vascular disease but just a persistently higher cardiac index; however there is no sufficient explanation as to why this could be established in cases but not in controls. Furthermore, cardiac output and PASP rapidly recover after exercise in a variable and non-proportional rate [25] and Argiento et al. have previously reported that this could be a reason why post-exercise measurements may be problematic [26]. However, in the latter study, PASP was estimated 5 to 20 minutes after exercise, while in the current study all measurements were conducted within 60 seconds. Although the method adopted in our study may still estimate PASP values less accurately than echocardiography during exercise [25], it has been previously used in order to assess pulmonary hypertension among scleroderma patients and was found to correlate well with several parameters of exercise capacity [27, 28].

In conclusion, pulmonary vascular disease, as established by high post exercise PASP, might be added to the list of determinants of both exercise impairment and increased dyspnea among CF patients with mild-to-moderate disease. To our knowledge, this study is the first to directly investigate the potential association between estimated PASP and maximal exercise capacity in these patients. The limitation in physical functioning and the increased dyspnea are the two primary parameters which affect quality of life in CF patients [29]. Under this scope, further studies, including a larger number of patients with different stages of disease severity are needed, in order for the contribution of pulmonary vascular disease in the physical impairment of this population to be fully evaluated.

## Figures and Tables

**Table 1:** Demographic characteristics, pulmonary function variables and pulmonary artery pressure measurements in cases and controls.

	Cases	Controls	*P* value
Number (M/F)	17 (11/6)	10 (7/3)	
Age (years)	23.9 ± 3.5	26.8 ± 3.1	NS
BMI (kg/m^2^)	21.3 ± 3.0	24.9 ± 4.1	NS
SS	72.3 ± 13.9	NA	
FEV_1_ (% predicted)	66.3 ± 24.3	99.1 ± 5.4	0.004
FVC (% predicted)	78.4 ± 18.6	98.5 ± 6.1	0.007
pO_2_ rest (mmHg)	75.7 ± 9.2	93.4 ± 9.2	0.008
SpO_2_ rest (%)	95.4 ± 1.9	97.6 ± 1.4	NS
PASP rest (mmHg)	27.5 ± 6.4	24.6 ± 2.2	NS
PASP post ex. (mmHg)	31.5 ± 7.3	25.8 ± 1.3	0.041
ΔPASP (mmHg)	4.0 ± 3.1	1.2 ± 1.3	0.048

Data are presented as mean ± 1 standard deviation. NS: not significant, BMI: body mass index, SS: Schwachmann score, NA: not applicable, rest: at rest, post ex.: post exercise, FEV_1_: forced expiratory volume in 1 second, FVC: forced vital capacity, pO_2_: arterial oxygen partial pressure, SpO_2_: oxygen saturation, PASP: right ventricular systolic pressure, ΔPASP: PASP post-PASP rest.

**Table 2:** Exercise parameters in cases and controls.

	Cases	Controls	*P* value
WR peak (% predicted)	66. ± 14.8	97.1 ± 5.3	0.002
VO_2_ peak (mL/kg∗min)	28.5 ± 7.5	35.9 ± 4.8	0.026
VO_2_ peak (% predicted)	73.7 ± 14.8	97.8 ± 5.9	0.002
AT (mL/kg∗min)	20.1 ± 5.4	27.1 ± 5.5	0.013
AT (% predicted)	48.8 ± 10.5	69.9 ± 12.3	0.008
VO_2_/HR (mL/kg∗beats)	10.8 ± 3.4	15.9 ± 4.9	0.032
VO_2_/HR (% predicted)	88 ± 17.2	98.1 ± 11.3	0.048
VE/VCO_2_@AT	31.3 ± 3.7	25.1 ± 3.0	0.008
VEmax (lit)	69.8 ± 21	81.1 ± 30.5	NS
VEmax/MVV (%)	66.7 ± 21.5	53.4 ± 18.1	NS
MVV-VEmax (lit)	40.01 ± 36.3	79.96 ± 34.9	0.047
BR (lit)	40 ± 36.3	79.7 ± 34.7	0.045
RER	1.19 ± 0.7	1.21 ± 0.6	NS
SpO_2_ peak (%)	93.9 ± 4	96 ± 1.9	NS
Borg scale fatigue (peak)	7.9 ± 1.4	8.2 ± 1.3	NS
Borg scale dyspnea (peak)	6.2 ± 2.9	5.9 ± 1.4	NS

Data are presented as mean ± 1 standard deviation. NS: not significant, WR peak: maximal work rate, VO_2_ peak: maximal oxygen uptake, AT: oxygen uptake at anaerobic threshold, VO_2_/HR: peak oxygen pulse, VE/VCO_2_@AT: ventilatory equivalent for carbon dioxide at anaerobic threshold, VE max: minute ventilation at peak exercise, VEmax/MVV: minute ventilation at peak exercise to maximum voluntary ventilation ratio, BR: breathing reserve, RER: respiratory exchange ratio, SpO_2_ peak: oxygen saturation at peak exercise.

**Table 3:** Exercise parameters in cystic fibrosis patients with postexercise PASP ≤35 mmHg and postexercise PASP >35 mmHg.

	PASP ≤35	PASP >35	*P* value
WR peak (watts)	135.7 ± 46.6	102.4 ± 30.1	NS
WR peak (% predicted)	73.6 ± 11.6	51.4 ± 7.5	0.008
VO_2_ peak (mL/kg∗min)	30.1 ± 8.0	25.1 ± 5.5	NS
VO_2_ peak (% predicted)	78.4 ± 15.4	63.4 ± 6.2	0.042
AT (mL/kg∗min)	20.3 ± 5.6	16.3 ± 1.6	NS
AT (% predicted)	51.9 ± 11.2	42.1 ± 4.6	NS
VO_2_/HR (mL/kg∗beats)	11.4 ± 3.8	9.2 ± 2	NS
VO_2_/HR (% predicted)	93.5 ± 17.6	75.9 ± 7.8	0.040
VE/VCO_2_@AT	28.1 ± 4.1	32.8 ± 2.3	0.061
VEmax (lit)	63.3 ± 21.6	57.5 ± 18.7	NS
VEmax/MVV (%)	64.3 ± 23.8	70.5 ± 14.9	NS
MVV-VEmax (lit)	39.6 ± 35.1	42.1 ± 38.9	NS
BR (lit)	42.8 ± 40.7	31.7 ± 21.2	NS
RER	1.19 ± 0.7	1.19 ± 0.4	NS
SpO_2_ peak (%)	95.4 ± 2.7	90.6 ± 4.7	0.016
Borg scale fatigue (peak)	7.9 ± 1.6	7.4 ± 0.6	NS
Borg scale dyspnea (peak)	5.9 ± 3.3	7.0 ± 2.2	0.046

Data are presented as mean ± 1 standard deviation. NS: not significant, WR peak: maximal work rate, VO_2_ peak: maximal oxygen uptake, AT: oxygen uptake at anaerobic threshold, VO_2_/HR: peak oxygen pulse, VE/VCO_2_@AT: ventilatory equivalent for carbon dioxide at anaerobic threshold, VEmax: minute ventilation at peak exercise, VEmax/MVV: minute ventilation at peak exercise to maximum voluntary ventilation ratio, BR: breathing reserve, RER: respiratory exchange ratio, SpO_2_ peak: oxygen saturation at peak exercise.

**Table 4:** Pearson correlations between ΔPASP and exercise variables in cases and controls.

	ΔPASP	
	Cases	Controls
WR peak (watts)	−0.539^∗^	NS
WR peak (% predicted)	−0.764^∗∗^	NS
VO_2_ peak (mL/kg∗min)	−0.540^∗^	NS
VO_2_ peak (% predicted)	−0.714^∗^	NS
AT (mL/kg∗min)	NS	NS
AT (% predicted)	NS	NS
VO_2_/HR (mL/kg∗beats)	−0.530^∗^	NS
VO_2_/HR (% predicted)	−0.663^∗∗^	NS
VE/VCO_2_@AT	NS	NS
SpO_2_ peak (%)	−0.701^∗^	NS

ΔPASP: PASP (pulmonary artery systolic pressure) after exercise − PASP at rest, WR peak: maximal work rate, VO_2_ peak: maximal oxygen uptake, AT: anaerobic threshold, VO_2_/HR: peak oxygen pulse, VE/VCO_2_@AT: ventilatory equivalent for carbon dioxide at anaerobic threshold, SpO_2_ peak: oxygen saturation at peak exercise; ^∗^ *P* < 0.05; ^∗∗^ *P* < 0.001; NS: not significant.

## References

[1] Moser C, Tirakitsoontorn P, Nussbaum E, Newcomb R, Cooper DM (2000). Muscle size and cardiorespiratory response to exercise in cystic fibrosis. *American Journal of Respiratory and Critical Care Medicine*.

[2] Shah AR, Gozal D, Keens TG (1998). Determinants of aerobic and anaerobic exercise performance in cystic fibrosis. *American Journal of Respiratory and Critical Care Medicine*.

[3] Pouliou E, Nanas S, Papamichalopoulos A (2001). Prolonged oxygen kinetics during early recovery from maximal exercise in adult patients with cystic fibrosis. *Chest*.

[4] Moorcroft AJ, Dodd ME, Morris J, Webb AK (2005). Symptoms, lactate and exercise limitation at peak cycle ergometry in adults with cystic fibrosis. *European Respiratory Journal*.

[5] Boutou AK, Pitsiou GG, Trigonis I (2011). Exercise capacity in idiopathic pulmonary fibrosis: the effect of pulmonary hypertension. *Respirology*.

[6] Fraser KL, Tullis DE, Sasson Z, Hyland RH, Thornley KS, Hanly PJ (1999). Pulmonary hypertension and cardiac function in adult cystic fibrosis: role of hypoxemia. *Chest*.

[7] Rovedder PME, Ziegler B, Pinotti AFF, Barreto SSM, Dalcin PDTR (2008). Prevalence of pulmonary hypertension evaluated by Doppler echocardiography in a population of adolescent and adult patients with cystic fibrosis. *Jornal Brasileiro de Pneumologia*.

[8] Florea VG, Florea ND, Sharma R (2000). Right ventricular dysfunction in adult severe cystic fibrosis. *Chest*.

[9] Vizza CD, Lynch JP, Ochoa LL, Richardson G, Trulock EP (1998). Right and left ventricular dysfunction in patients with severe pulmonary disease. *Chest*.

[10] Ionescu AA, Payne N, Obieta-Fresnedo I, Fraser AG, Shale DJ (2001). Subclinical right ventricular dysfunction in cystic fibrosis: a study using tissue Doppler echocardiography. *American Journal of Respiratory and Critical Care Medicine*.

[11] Eckles M, Anderson P (2003). Cor pulmonale in cystic fibrosis. *Seminars in Respiratory and Critical Care Medicine*.

[12] American Thoracic Society (1995). Standardization of spirometry (1994 update). *American Journal of Respiratory and Critical Care Medicine*.

[13] Galiè N, Hoeper MM, Humbert M (2009). Guidelines for the diagnosis and treatment of pulmonary hypertension. *European Respiratory Journal*.

[14] Schiller NB (1991). Two-dimensional echocardiographic determination of left ventricular volume, systolic function, and mass. Summary and discussion of the 1989 recommendations of the American Society of Echocardiography. *Circulation*.

[15] Wasserman K, Hansen JE, Sue DY, Stringer WW, Whipp BJ, Wasserman K, Hansen JE, Sue DY, Stringer WW, Whipp BJ (2005). Measurements during integrative cardiopulmonary exercise testing. *Principles of Exercise-Testing and Interpretation*.

[16] American Thoracic Society/American College of Chest Physicians (2001). Statement on cardiopulmonary exercise testing. *American Journal of Respiratory and Critical Care Medicine*.

[17] Montgomery GS, Sagel SD, Taylor AL, Abman SH (2006). Effects of sildenafil on pulmonary hypertension and exercise tolerance in severe cystic fibrosis-related lung disease. *Pediatric Pulmonology*.

[18] Henno P, Maurey C, Danel C (2009). Pulmonary vascular dysfunction in endstage cystic fibrosis: role of NF-*κ*B and endothelin-1. *European Respiratory Journal*.

[19] Rovedder PME, Ziegler B, Pasin LR (2007). Doppler echocardiogram, oxygen saturation and submaximum capacity of exercise in patients with cystic fibrosis. *Journal of Cystic Fibrosis*.

[20] Ting H, Sun XG, Chuang ML, Lewis DA, Hansen JE, Wasserman K (2001). A noninvasive assessment of pulmonary perfusion abnormality in patients with primary pulmonary hypertension. *Chest*.

[21] Sun XG, Hansen JE, Oudiz RJ, Wasserman K (2001). Exercise pathophysiology in patients with primary pulmonary hypertension. *Circulation*.

[22] Gea J, Casadevall C, Pascual S, Orozco-Levi M, Barreiro E (2012). Respiratory diseases and muscle dysfunction. *Expert Review of Respiratory Medicine*.

[23] Lands LC, Heigenhauser GJF, Jones NL (1992). Analysis of factors limiting maximal exercise performance in cystic fibrosis. *Clinical Science*.

[24] Homma A, Anzueto A, Peters JI (2001). Pulmonary artery systolic pressures estimated by echocardiogram vs cardiac catheterization in patients awaiting lung transplantation. *Journal of Heart and Lung Transplantation*.

[25] Naeije R (2011). In defence of exercise stress test for the diagnosis of pulmonary hypertension. *Heart*.

[26] Argiento P, Chesler N, Mulè M (2010). Exercise stress echocardiography for the study of the pulmonary circulation. *European Respiratory Journal*.

[27] Steen V, Chou M, Shanmugam V, Mathias M, Kuru T, Morrissey R (2008). Exercise-induced pulmonary arterial hypertension in patients with systemic sclerosis. *Chest*.

[28] Alkotob ML, Soltani P, Sheatt MA (2006). Reduced exercise capacity and stress-induced pulmonary hypertension in patients with scleroderma. *Chest*.

[29] de Jong W, Kaptein AA, van der Schans CP (1997). Quality of life in patients with cystic fibrosis. *Pediatric Pulmonology*.

